# Morphofunctional Characterization of Different Tissue Factors in Congenital Diaphragmatic Hernia Affected Tissue

**DOI:** 10.3390/diagnostics11020289

**Published:** 2021-02-12

**Authors:** Ricards Kaulins, Laura Ramona Rozite, Mara Pilmane, Aigars Petersons

**Affiliations:** 1 Institute of Anatomy and Anthropology, Riga Stradins University, 9 Kronvalda Boulevard, LV-1010 Riga, Latvia; rozitesr@gmail.com (L.R.R.); Mara.Pilmane@rsu.lv (M.P.); 2 Department of Pediatric Surgery, Children’s Clinical University Hospital, 45 Vienibas gatve Street, LV-1004 Riga, Latvia; aigars.petersons@rsu.lv; 3 Department of Pediatric Surgery, Riga Stradins University, 16 Dzirciema Street, LV-1007 Riga, Latvia

**Keywords:** tissue factors, congenital hernia, diaphragm, neonates

## Abstract

Congenital diaphragm hernia (CDH) is a congenital disease that occurs during prenatal development. Although the morbidity and mortality rate is rather significant, the pathogenesis of CDH has been studied insignificantly due to the decreased accessibility of human pathological material. Therefore the aim of our work was to evaluate growth factors (transforming growth factor-beta (TGF-β), basic fibroblast growth factor (bFGF), insulin-like growth factor 1 (IGF-1), hepatocyte growth factor (HGF)) and their receptors (fibroblast growth factor receptor 1 (FGFR1), insulin-like growth factor 1 (IGF-1R)), muscle (dystrophin, myosin, alpha actin) and nerve quality (nerve growth factor (NGF), nerve growth factor receptor (NGFR), neurofilaments (NF)) factors, local defense factors (ß-defensin 2, ß-defensin 4), programmed cell death (TUNEL), and separate gene (Wnt-1) expression in human pathological material to find immunohistochemical marker differences between the control and the CDH patient groups. A semi-quantitative counting method was used for the evaluation of the tissues and structures in the Biotin-Streptavidin-stained slides. Various statistically significant differences were found in immunoreactive expression between the patient and the control group tissue and the morphological structures as well as very strong, strong, and moderate correlations between immunoreactives in different diaphragm cells and structures. These significant changes and various correlations indicate that multiple morphopathogenetic pathways are affected in CDH pathogenesis. This work contains the evaluation of the causes for these changes and their potential involvement in CDH pathogenesis.

## 1. Introduction

Congenital diaphragmatic hernia (CDH) is a developmental defect with the incidence in Europe estimated to be 2.3 per 10.000 births [1]. Multiple embryonic tissue sources are involved in the development of a functional diaphragm forming blood vessels, muscle cells, and connective tissue [2]. The tissues necessary for diaphragm development are mainly supported by four embryonic components: septum transversum, pleuro-peritoneal folds (PPF), dorsal mesentery of esophagus, and muscular ingrowths from lateral body walls. During prenatal development, these structures may fail to fuse thereby resulting in formation of CDH. The contents of the abdominal cavity herniate into the thoracic cavity through the defected diaphragm because of the pressure gradient between the cavities thus causing pulmonary hypoplasia, pulmonary hypertension, and heart failure resulting in high mortality rates.

CDH is an etiologically heterogeneous developmental defect. However, the genes involved in pathogenesis are mainly unknown, Alaggio et al. in their research state that the concerted action of GATA-4, Fog2, COUP-TFII and WT-1 [3] is required for a proper development of the diaphragm. GATA-4, COUP-TFII, and WT-1 significance in CDH development is intertwined with the retinoic acid signaling pathway. *GATA-4* activation and expression are both influenced by retinoids. *COUP-TFII* is a downstream target of retinoid signaling, and *WT-1* gene is responsible for encoding a zinc-finger-containing protein which is involved in the retinoic acid signaling pathway. The retinoic acid signaling pathway is crucial for normal pre- and post-natal development being involved in the specification and formation of many organs and tissues one of which is a proper diaphragm development [4]. The transcription factors GATA-4, COUP-TFII and Fog2 are expressed in early development of PPFs [5].

Although the morbidity and mortality rate of the patients with CDH is significant, the pathogenesis of CDH has been studied insufficiently due to the decreased accessibility of human pathological material. Growth factors and their receptors, muscle and nerve quality, local defense factor, programmed cell death, and separate gene markers could reveal their crucial role in the development of CDH.

Basic fibroblast growth factor (bFGF) manifests broad mitogenic and cell survival activities. It plays an important role in a variety of biologic processes including embryonic development, cell growth morphogenesis, tissue repair, tumor growth, survival, proliferation, apoptosis and invasion [6]. bFGF influences mitosis by inhibiting phosphorylation of translation initiation factor, which is crucial for cell progression [7]. bFGF can activate fibroblast growth factor receptor (FGFR) 1c, 1b, 2c, 3c and 4 thus initiating a tyrosine kinase signaling pathway that has a fundamental role in many biologic processes including embryonic development, tissue regeneration, and angiogenesis. It also plays important roles in diverse cell functions, including proliferation, differentiation, apoptosis and migration [6,8]. bFGF, via activation of FGFR1, is a highly potent inducer of angiogenesis. It can even be twice as potent as vascular endothelial growth factor (VEGF). It is hypothesized that there is a crosstalk between members of the VEGF family and bFGF during angiogenesis due to their synergetic effect [9]. 

Transforming growth factor-beta (TGF-β) is a pleiotropic cytokine involved in development, various cellular responses and homeostasis of most human tissues [10]. Activation of TGF-beta receptors affects gene expression through activation of SMAD family transcription factors, thus regulating cell proliferation, differentiation and growth and it can modulate expression and activation of other growth factors [11,12]. For example, TGF-β1 has a significant inhibitory effect on hepatocyte growth factor (HGF) mRNA expression; thus, the decrease in the fibroblast size leads to the up-regulation of the HGF expression [13]. The production of numerous extracellular matrix proteins and inhibition of degrading of these proteins is stimulated by TGF-β [10]. TGF-β influences angiogenesis by various mechanisms; for instance, by alternating two signaling cascades with opposite results (ALK1 and ALK5); thereby it is involved in vessel proliferation and maturation. TGF-β can also stimulate its own expression and control the expression of other angiogenic factors, such as platelet-derived growth factor, interleukine-1, bFGF, tumor necrosis factor alpha and transforming growth factor alpha [14].

Insulin-like growth factor 1 (IGF-1) is an insulin-like protein with a similar function and structure involved in mediating growth and development [15]. IGF-1 is the major mediator of prenatal and post-natal growth [16]. IGF-1 exerts multiple physiological and pathophysiological effects on the vasculature including hypertrophic, survival, vasomotor and metabolic effects via its receptor (IGF-1R), stimulating smooth muscle cell proliferation, migration and inhibiting smooth muscle cell apoptosis through both endocrine and autocrine/paracrine mechanisms [17,18]. The effects of IGF-1 are mediated principally through IGF-1R, but they are modulated by complex interactions with multiple IGF binding proteins that are regulated by kinase activation, proteolysis, polymerization and the initiation of intracellular signaling via the AKT signaling pathway [18]. The signaling mechanism of IGF-1/PI3-kinase/Akt plays an important role in controlling the angiogenic phenotype through the activation of angiogenic growth factors to induce autocrine PI3-kinase/Akt signaling in endothelial cells [19].

Hepatocyte growth factor (HGF) is a central growth factor that modulates proliferation, angiogenesis, morphogenesis, motility and regulates cell growth [13,20]. HGF acts as a morphogen in embryonic development and promotes cell migration. Muscle cells migrate from somites into the PPFs in the presence of HGF. It is expressed along the migratory path of migratory muscle precursors, including the PPFs. The muscle precursors will then spread to all regions of mesenchymal cells as well as endothelium and non-hepatocyte liver cells [21]. This protein also plays a role in angiogenesis, tumorigenesis and tissue regeneration [22].

Furthermore, skeletal muscle fibers display distinct quality markers such as myosin, dystrophin, and alpha actin. We can speculate about loss of muscle mass, muscle atrophy as well as quality of muscle tissue through these factors.

Nerve growth factor (NGF) is expressed in developing and differentiating sympathetic and sensory neurons [23]. NGF can bind to tyrosine kinase receptors (TrkA) inhibiting apoptosis and to p75 nerve growth factor receptors (p75NGFR) in which case it stimulates sensory neuronal cell growth and differentiation, but it might also trigger apoptosis [23]. NGFR is also located at sites that do not have access to NGF [24]. NGFR are found along the path of the neuronal cell migration from the neural tube to the PPFs and thus may lead to the outgrowth of the phrenic nerve [2]. No effect of NGF on motor neurons is known [25], although proNGF (the precursor form of NGF) promotes apoptosis of motor neurons by binding to p75NGFR [26]. Myoblast cells produce p75NGFR during myogenesis. NGF is expressed adjacently to the developing muscle fibers. Once the myotubes have formed, NGF and p75NGFR cease to be expressed [26].

Neurofilaments (NF) are a part of the mature neuronal cytoskeleton and the quality marker of the neuronal structures. They are essential in nervous system development, neuronal regeneration and electrical impulse transmission [27].

Human beta defensins (β-defensin) are a part of an organism’s defense mechanism; therefore, in normal conditions they are not expressed in great amount, but their expression is triggered by inflammation, microbial pathogenicity factors, tissue damage, and other host factors. They are released primary by epithelial cells as well as by leukocytes, skeletal muscle, heart and other organs [28]. According to the study of Baroni et al. β-defensin 2 can stimulate chemotaxis of human endothelial cells to a similar degree of VEGF [29]. β-defensin 4 exhibits anti-microbial activity, but it does not respond to inflammatory markers: interleukin 6 (IL-6), interferon γ (IFNγ), tumor necrosis factor α (TNF-α) and IL-1. It is also expressed in neutrophils and it attracts monocytes as well [30].

Terminal deoxynucleotidyl transferase (TdT) dUTP Nick-End Labeling (TUNEL) assays are used to detect DNS fragmentation during apoptosis or sometimes DNA destruction from genotoxic agents [31]. The labeling of free 3′-OH ends in DNA is catalyzed by the TdT enzyme to prevent the cross-linking of free ends [31]. Nitrofen-exposed rat models show opposing results: some show that CDH has formed due to decreased cell proliferation but others voice an opinion that the increased apoptosis has been the cause of CDH [32].

Wnt-1 is a wingless class signaling protein that induces a pathway, which leads to an increased expression of genes that are important in regulation of cell death, proliferation, migration and patterning in embryonic development by inhibiting or activating the frizzled receptors [33]. During early organogenesis, genes associated with Wnt signaling in the diaphragm were shown to be preferentially expressed, and a few Wnt pathway members have been associated with CDH [34].

Based on the mentioned above, our aim was the detection of appearance and distribution of different growth factors and their receptors, muscle and nerve quality factors, local defense factors, programmed cell death, and separate gene expression in the different diaphragm sites of diaphragm hernia patients and comparison of the results with the control group in order to find those changes that could lead to the morphopathogenetic mechanism in the development of CDH.

## 2. Materials and Methods

### 2.1. Materials Characteristics of Subjects

The research work was done in accordance with the Helsinki declaration. The study was approved by the Ethical Committee at Riga Stradins University, the permit was issued on 10 May 2007. The diaphragm material was obtained from 5 children (2 boys and 3 girls) age from 1 to 2 days. The patient group represented 4 children with CDH, and the control group represented the intact part of the diaphragm of a patient with CDH and one child without pathology in the diaphragm. All the examined patients’ hernias were localized posterolaterally, where the mean size of hernias was 4–6 × 2–3 cm. Four necropsies of CDH from the margin of diaphragm hernia and two control necropsies from 3 different diaphragm sites were obtained from neonates 12 h after their death at Children Clinical University Hospital. The localizations of diaphragm necropsy sites were proximal—near the longitudinal axis, distal—near the body wall, central—in between proximal and distal sites. The compiled material is the property of the Institute of Anatomy and Anthropology in Riga Stradins University.

### 2.2. Immunohistochemical Analysis

The samples were fixed in a mixture of 2% formaldehyde and 0.2% picric acid in 0.1 M phosphate buffer (pH 7.2) for 24 h. Then the samples were rinsed for 12 h in Tyrode buffer (content: NaCl, KCl, CaCl_2_ ·2H_2_O, MgCl_2_·6H_2_O, NaHCO_3_, NaH_2_PO_4_·H_2_O, glucose) containing 10% saccharose. Afterwards the samples were embedded into paraffin. Three micrometers thin sections had been cut; they were then stained with hematoxylin and eosin for routine morphological evaluation. For immunohistochemistry (IMH) Biotin-Streptavidin biochemical method was used to detect: TGF-β (orb77216, working dilution 1:100, Biorbyt Limited, Cambridge, UK), bFGF (ab16828, working dilution 1:200, Abcam, Cambridge, UK), FGFR1 (orb38277, working dilution 1:100, Biorbyt Limited, UK), IGF-1 (MAB291, working dilution 1:50, RD systems, McKinley Place NE, MN, USA), IGF-1R (AF-305-NA, working dilution 1:100, RD systems, USA), HGF (AF-294-NA, working dilution 1:200, RD systems, USA), NGF (ab6199, working dilution 1:500, abcam, UK), NGFR (sc-56448, working dilution 1:150, Santa Cruz Biotechnology, Inc., Dallas, TX, USA), myosin (ab7784, working dilution 1:150, abcam, UK), dystrophin (ab15277, working dilution 1:100, abcam, UK), alpha actin (α-actin, HHF35, working dilution 1:100, Cell Marque Corporation, USA), wingless gene 1 (Wnt-1, ab15251, working dilution 1:100, abcam, UK), beta defensin 2 (ß-defensin 2, sc-20798, working dilution 1:100, Santa Cruz Biotechnology, Inc., Dallas, TX, USA), beta defensin 4 (ß-defensin 4, ab14419, working dilution 1:150, abcam, UK), NF (2F11, working dilution 1:100, Cell Marque Corporation, USA). TUNEL, In Situ Cell Death Detection, POD (1684817, Roche, Indianapolis, IN, USA), DAB Substrate Vector Kit (SK4100, Vector Laboratories, Burlingame, CA, USA), was used to reveal apoptosis.

A semi-quantitative counting method was used for the evaluation of the tissues and structures in the stained slides. Tissue and morphological structures were graded by the appearance of positively stained cells in the visual field. The used designations were as follows: 0—no positive structures, 0/+: occasional positive structures, +: few positive structures, +/++: few to moderate number of positive structures, ++: moderate number of positive structures, ++/+++: moderate to numerous positive structures, +++: numerous positive structures, +++/++++: numerous to abundant structures, ++++: abundance of positive structures in the visual field [35].

The stained slides were evaluated using Leica DC 300F digital camera and image processing and analysis software Image Pro Plus (Media Cybernetics, Inc., Rockville, MD, USA).

### 2.3. Statistical Analysis

The data processing was conducted using Statistical Package for the Social Sciences (SPSS) program version 20.0. The results from semi-quantitative evaluated tissue and structures were transformed into numerical form as follows: 0: equals to 0, 0/+: equals to 0.5, +: equals to 1, +/++: equals to 1.5, ++: equals to 2, ++/+++: equals to 2.5, +++: equals to 3, +++/++++: equals to 3.5, ++++: equals to 4. The test of normal distribution showed that data was not distributed normally, thus nonparametric statistics was used. Nonparametric Mann–Whitney U test was used to determine statistically significant differences between the patient and the control groups and Spearman’s rank correlation coefficient was calculated to detect correlations between the factors in the patient group, where r = 0–0.19 was assumed as a very weak correlation, r =0.2–0.39—weak correlation, r = 0.4–0.59—moderate correlation, r = 0.6–0.8—strong correlation and r = 0.8–1.0—a very strong correlation. For all the tests *p*-value was chosen 5% (*p*-value < 0.05) as statistically significant.

## 3. Results

### 3.1. Tissue Review

**Figure 1:**
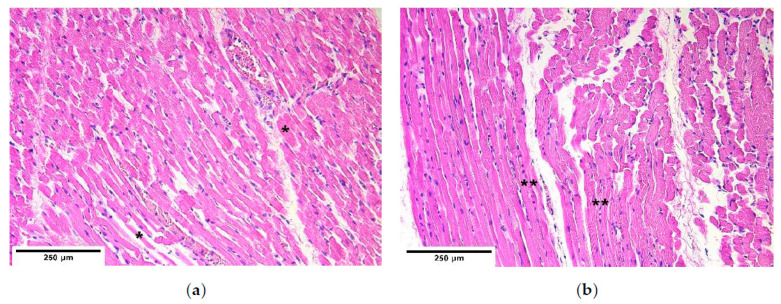
Micrographs of diaphragm tissue. (**a**) Note the different diameters of muscle fiber size (*). Hematoxylin and eosin; (**b**) visible immature muscle fibers (**). Hematoxylin and eosin, ×200.

Different fiber diameter, perinuclear vacuolisation and newly formed muscle fibers were present in both the control and the patient groups (Figure 1a,b). In some cases, sclerotic arterioles were observed in the perimysium.

### 3.2. Immunohistochemical (IMH) Data

**Figure 2:**
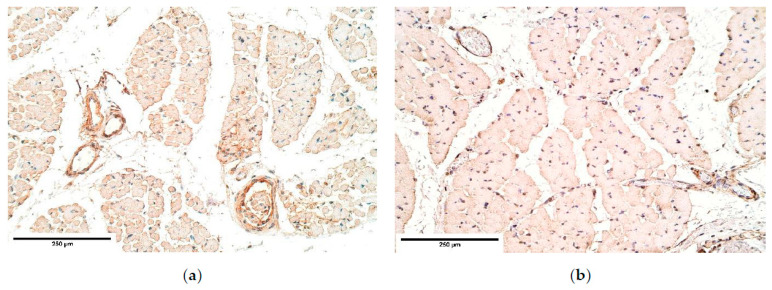
(**a**) A moderate number of positive TGF-ß structures in a CDH patient. TGF-ß IMH; (**b**) A moderate number of positive TGF-ß weakly stained muscle fibers in the control. TGF-ß IMH, ×200. TGF-β—Transforming growth factor-beta; IMH—immunohistochemistry.

TGF-ß was observed in all tissue samples. TGF-ß presented moderate expression across different diaphragm sites (proximal, central and distal) showing no differences between the patient and the control group muscle fibers (Table 1, Figure 2a,b). Upon viewing TGF-ß in blood vessels, a few to moderate number of factor positive structures were noted in the blood vessel wall. Connective tissue demonstrated a few positive cells in the proximal diaphragm; however, the central and the distal parts of the diaphragm showed fluctuations of TGF-ß expression (from almost no to moderate expression). Moderate number of TGF-ß positive mesotheliocytes was found in the proximal and the distal parts of the diaphragm, while the central (herniated) part showed only a few positive cells in the patient group whereas an increase in positive structures was noted in the control group (a moderate number of positive structures) (Table 1).

**Figure 3:**
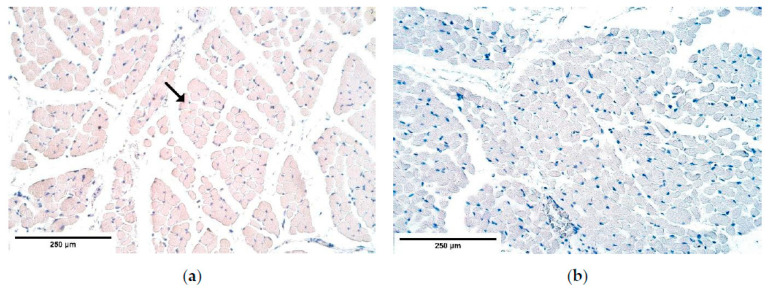
(**a**) Occasional bFGF positive muscle fibers in a CDH patient. Arrow: occasional bFGF positive muscle fibers in primary muscle bundle of the diaphragm. bFGF IMH; (**b**) An absence of bFGF in the control. bFGF IMH, ×200. bFGF—basic fibroblast growth factor; IMH—immunohistochemistry.

bFGF was almost absent from the diaphragm tissue only marking occasional muscle fibers; however, bFGF showed fluctuations from occasional positive muscle fibers to an abundance of bFGF positive muscle fibers (Table 1, Figure 3a,b) in the distal part of the CDH affected diaphragm.

**Figure 4:**
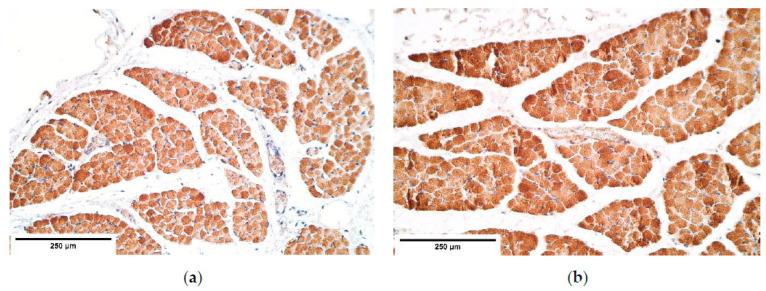
(**a**) Numerous to abundant FGFR1 positive structures in a CDH patient. FGFR1 IMH; (**b**) Abundance of FGFR1 positive structures in the control group tissue. FGFR1 IMH, ×200. FGFR1—Fibroblast growth factor receptor 1; IMH—immunohistochemistry.

FGFR1 presented a stable expression both in the control and the patient groups muscle fibers; however, upon comparing these groups, it was found that FGFR1 was slightly more expressed in the control group muscle fibers (abundance of FGFR1 positive structures) than in the patient group muscle fibers (from moderate to numerous FGFR1 positive structures). High fluctuations were noted in the proximal and the distal diaphragm connective tissue variating from occasional to numerous positive cells (Table 1, Figure 4a,b), while the central (herniated) part displayed the number of positive cells that varied from occasional to moderate in both the patient and the control groups. The evaluation of blood vessels demonstrated moderate to numerous positive FGFR1 structures throughout the diaphragm sites. Numerous FGFR1 positive mesotheliocytes were found in the proximal and the central parts of the diaphragm whereas the distal part had occasional FGFR1 positive mesotheliocytes (Table 1).

**Figure 5:**
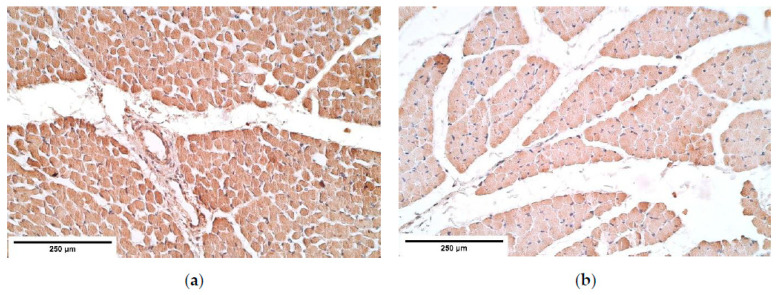
(**a**) Numerous IGF-1 positive structures in a CDH patient. IGF IMH; (**b**) Moderate to numerous IGF-1 positive structures in the control group tissue. IGF IMH, ×200. IGF-1—Insulin-like growth factor 1; IMH—immunohistochemistry.

IGF-1 presented the most variable expression in muscle fibers. The proximal part of the CDH affected diaphragm showed a few positive muscle fibers, whereas the controls possessed numerous positive muscle fibers. The central part demonstrated an increase of IGF-1 positive muscle fibers (moderate to numerous positive muscle fibers) in the patient group but a few to numerous positive muscle fibers were found in the control group (Table 1, Figure 5a,b). The distal part of the CDH affected muscle fibers showed an increased IGF-1 expression (moderate to numerous positive muscle fibers), whereas the control group had a notable decrease (few positive muscle fibers) (Table 2).

**Figure 6:**
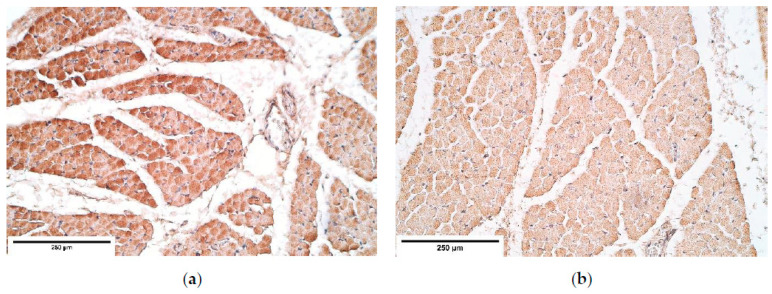
(**a**) Moderate to numerous IGF-1R positive structures in a CDH patient. IGF-1R IMH; (**b**) Numerous IGF-1R positive weakly stained structures in the control group tissue. IGF-1R IMH, ×200. IGF-1R—Insulin-like growth factor 1 receptor; IMH—immunohistochemistry.

On average moderate to numerous IGF-1R positive muscle fibers, numerous positive connective tissue, moderate to numerous positive blood vessels and numerous factor positive mesotheliocytes were observed in both the CDH patient and the control groups (Table 2, Figure 6a,b).

**Figure 7:**
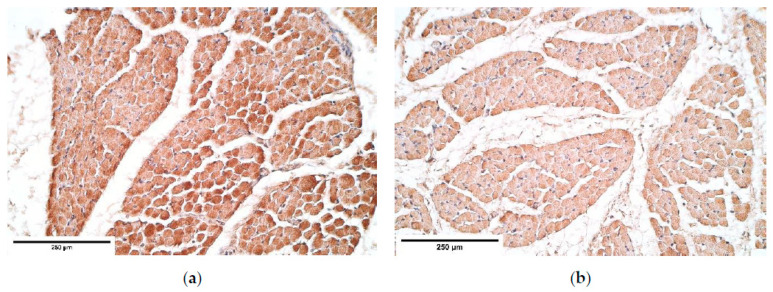
(**a**) Moderate to numerous HGF positive muscles in a CDH patient. HGF IMH; (**b**) Moderate to numerous HGF positive weakly stained structures in the control group tissue. HGF IMH, ×200. HGF—Hepatocyte growth factor; IMH—immunohistochemistry.

HGF immunoreactive structures were revealed in a very stable appearance—from moderate to numerous positive muscle fibers and blood vessels, and numerous positive connective tissue and mesothelial cells (Table 2, Figure 7a,b)

**Figure 8:**
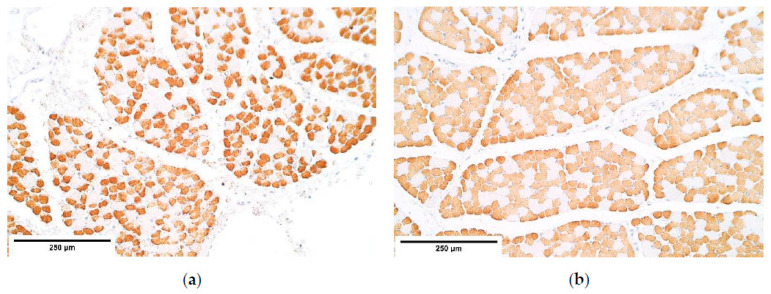
(**a**) Moderate number of myosin positive intensively stained muscle fibers in a CDH patient. Myosin IMH; (**b**) Moderate to numerous positive myosin muscle fibers in the control group tissue. Myosin IMH, ×200. IMH—immunohistochemistry.

Myosin in the controls was expressed in all the diaphragm parts equally showing moderate to numerous positive muscle fibers. The patient tissue revealed a similar expression in the proximal and the central parts of CDH; while the distal part of the diaphragm demonstrated the fluctuation from few to numerous positive muscle fibers (Table 3, Figure 8a,b).

**Figure 9:**
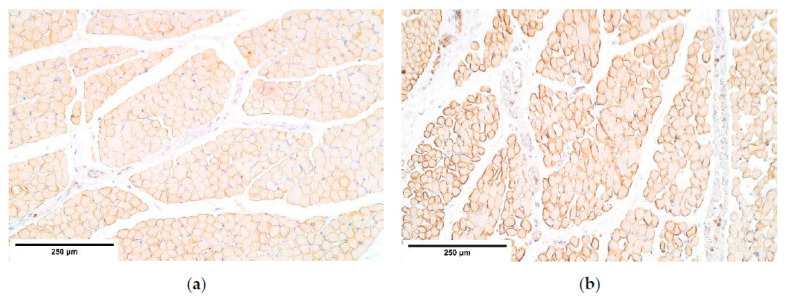
(**a**) Moderate number of dystrophin positive muscles in CDH. Dystrophin IMH; (**b**) Moderate number of positive dystrophin muscles in the control group tissue. Dystrophin IMH, ×200. IMH—immunohistochemistry.

Dystrophin presented from few to numerous positive muscle fibers in the proximal part of CDH affected diaphragm, while a moderate number of muscle fibers was seen in the central diaphragm. The distal part of the CDH affected diaphragm presented from occasional to numerous dystrophin positive muscle fibers, whereas the control group showed consistent distribution from few to numerous positive dystrophin muscle fibers (Table 3, Figure 9a,b).

**Figure 10:**
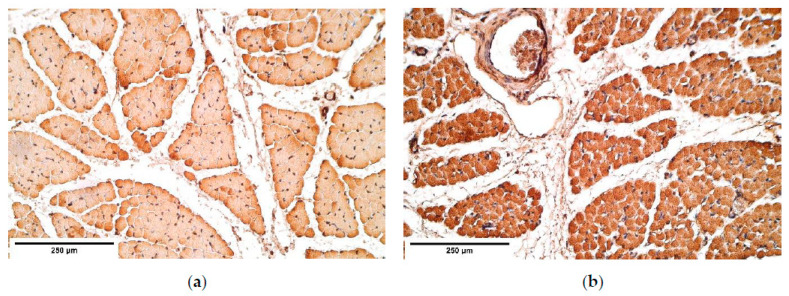
(**a**) Moderate to numerous positive α-actin muscle fibers in a CDH patient. α-actin IMH; (**b**) Numerous to abundant α-actin muscle fibers in the control group tissue. α-actin IMH, ×200. IMH—immunohistochemistry.

α-actin expression was relatively stable with numerous positive muscle fibers and an abundance of positive blood vessels in the CDH and the control diaphragms (Table 3, Figure 10a,b).

**Figure 11:**
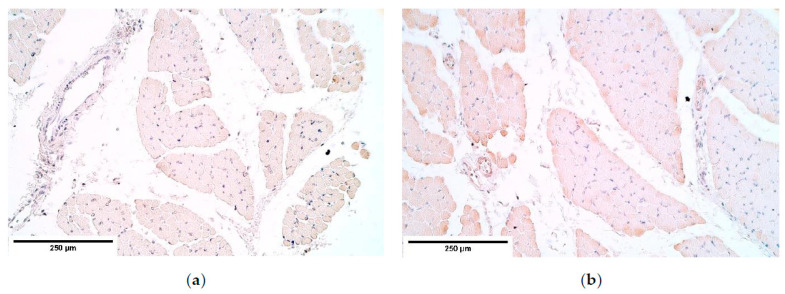
(**a**) Few β-defensin 2 positive muscle fibers in CDH. β-defensin 2 IMH; (**b**) Moderate β-defensin 2 positive muscle fibers in a patient without CDH. β-defensin 2 IMH, ×200. IMH—immunohistochemistry.

Evaluating β-defensin 2 appearance in the diaphragm tissue, β-defensin 2 expression was relatively stable, showing a moderate number of positive muscle fibers, occasional positive connective tissue, and few positive endotheliocytes in the CDH and the controls. However, mesotheliocytes presented a variable expression. β-defensin 2 was completely absent in the central part of the CDH affected diaphragm; however, an increase of positive factor structures (up to numerous positive mesotheliocytes) was found in the control group (Table 4, Figure 11a,b).

**Figure 12:**
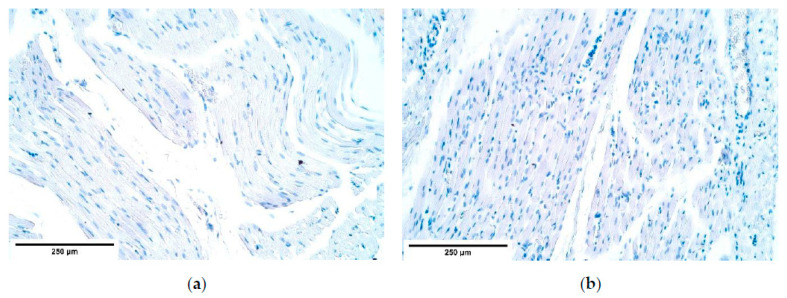
The lack of β-defensin 4 positive structures in a CDH patient (**a**) and in the control group (**b**). β-defensin 4 IMH, ×200. IMH—immunohistochemistry.

β-defensin 4 showed occasional positive muscle fibers only in the central part of the diaphragm yet was absent everywhere else (Table 4, Figure 12a,b).

**Figure 13:**
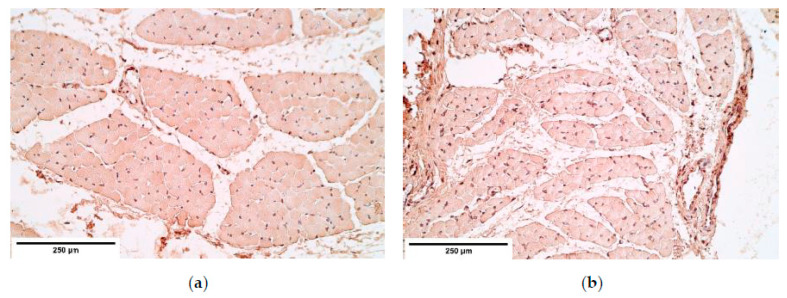
(**a**) A few apoptotic connective tissue cells in a patient with CDH. TUNEL; (**b**) Moderate apoptotic nuclei in the control group muscle fibers, connective tissue, and endothelium. TUNEL ×200.

Apoptosis affected a moderate number of muscle fiber nuclei in both the patient and the control groups. However, the number of apoptotic connective tissue cells and endotheliocytes varied from few to numerous in the CDH affected tissue; the control group showed a stable number of moderate to numerous positive structures. The number of apoptotic mesotheliocytes fluctuated from few positive structures to an abundance of positive structures both in the patient and the control groups (Table 4, Figure 13a,b).

**Figure 14:**
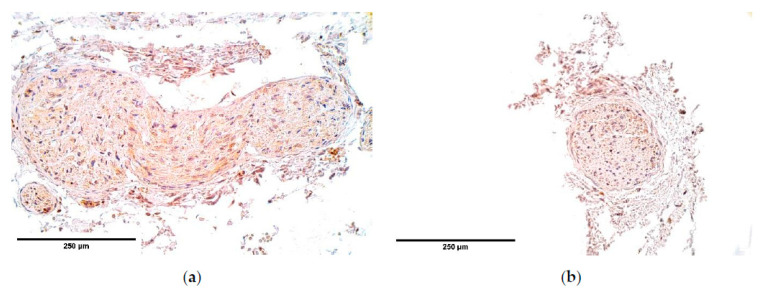
(**a**) Moderate expression of NGF in a nerve bundle in a patient with CDH. NGF IMH; (**b**) Moderate distribution of NGF in a nerve bundle and among the muscle fibers in the control group tissue. NGF IMH, ×250. NGF—nerve growth factor; IMH—immunohistochemistry.

NGF marked only a few nerves among the muscle fibers, with a slight increase (few to moderate number of positive structures) in the distal diaphragm of CDH cases. On the contrary, the number of blood vessels and solitarily located NGF positive nerve bundles were always higher in the control group in comparison to the CDH affected tissue (Table 5, Figure 14a,b).

**Figure 15:**
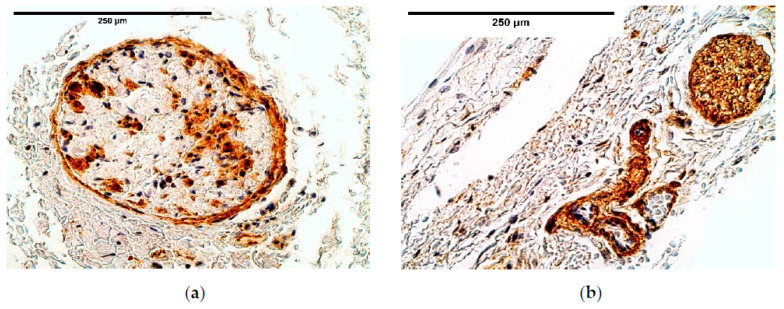
(**a**) Moderate NGFR-containing nerve fibers in a CDH patient. NGFR IMH; (**b**) Moderate to numerous NGFR positive nerves in the control group tissue. NGFR IMH, ×400. NGFR—nerve growth factor receptor; IMH—immunohistochemistry.

We found relatively small differences between the patient and the control groups in NGFR positive structures, where the expression in the connective tissue was fluctuating from occasional to few positive cells. NGFR positive nerve fibers among the blood vessels and those in nerve bundles had notable variations from absence of positive structures to numerous positive structures with a tendency of lower expression in the CDH affected tissue than in the control group (Table 5, Figure 15a,b).

**Figure 16:**
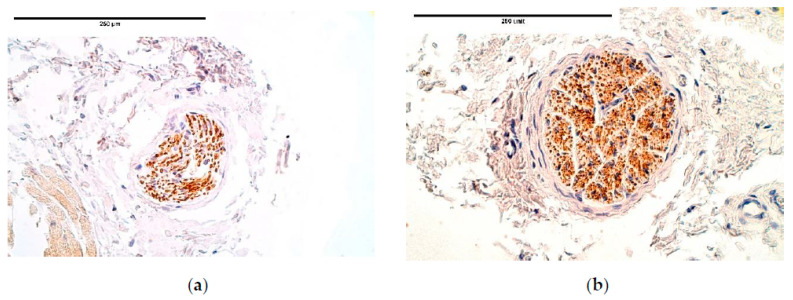
(**a**) Moderate NF nerve fibers in connective tissue of the CDH diaphragm. NF IMH; (**b**) Moderate to numerous NF-containing nerve fibers in the control group tissue. NF IMH, ×400. NF—neurofilaments; IMH—immunohistochemistry.

The variable expression from total absence to numerous NF positive nerve fibers was detected in the central diaphragm both in the patients and the controls; however, no NF positive nerves were found in the patient proximal and distal parts of the diaphragm, while fluctuations of NF remained equal in the control group—from total absence to numerous positive structures (Table 5, Figure 16a,b).

**Figure 17:**
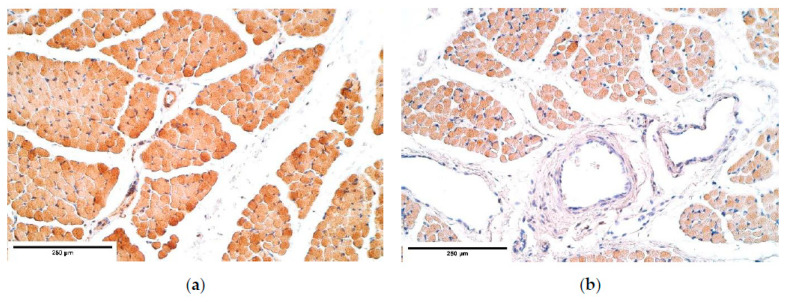
(**a**) Numerous Wnt-1 positive intensively stained muscle fibers in a CDH patient. Wnt-1 IMH; (**b**) Numerous Wnt-1 positive muscle fibers in the control group tissue. Wnt-1 IMH, ×200. IMH—immunohistochemistry.

Wnt-1 demonstrated a stable expression of numerous to abundant positive muscle fibers and mostly no positive structures in the connective tissue. However, it was found that Wnt-1 fluctuated significantly from total absence to abundance of positive endotheliocytes and mesotheliocytes in the CDH affected diaphragm tissue whereas the control group presented a stable expression (Table 6, Figure 17a,b).

### 3.3. Statistical Analysis

Statistically significant differences were found between the patient and the control group tissue and morphological structures. bFGF (Mann–Whitney *U*: 10; Z-score: −2.592; *p*-value: 0.013) and FGFR1 (Mann–Whitney U: 9; Z-score: −2.598; *p*-value: 0.01) immunoreactives showed significant changes between the patient and the control group muscle fibers. TGF-β (Mann–Whitney U: 13.5; Z-score: −2.399; *p*-value: 0.032), FGFR1 (Mann–Whitney *U*: 7; Z-score: −2.781; *p*-value: 0.005), IGF-1R (Mann–Whitney *U*: 9; Z-score: −2.797; *p*-value: 0.01), β-defensin 2 (Mann–Whitney *U*: 14.5; Z-score: −2.125; *p*-value: 0.041), TUNEL (Mann–Whitney *U*: 14.5; Z-score: −2.063; *p*-value: 0.041) and NGF (Mann–Whitney *U*: 10.5; Z-score: −2.485; *p*-value: 0.013) immunoreactives revealed significant changes between the patient and the control group blood vessels. Only HGF (Mann–Whitney U: 0; Z-score: −4.123; *p*-value: < 0.001) was found statistically significant in connective tissue between the groups. No statistically significant data was found in mesothelium.

Spearman’s rank correlation coefficient was used to detect correlations between immunoreactives in the patient group. A very strong positive correlation was detected between TGF-β and FGFR1, a strong positive correlation between myosin and TUNEL, HGF and IGF-1R, FGFR1 and IGF-1, bFGF and FGFR1, TGF-β and IGF-1, bFGF and IGF-1R, IGF-1 and bFGF, IGF-1 and β-defensin 4, whereas a moderate positive correlation was detected between TGF-ß and bFGF in the muscle fibers (Table 7).

A very strong positive correlation was detected between FGFR1 and IGF1, a strong positive correlation between HGF and TGF-β, Wnt-1 and IGF-1, Wnt-1 and IGF-1R, Wnt-1 and FGFR1, TGF-ß and β-defensin 2, TGF-β and FGFR1 TGF-β and IGF-1, IGF-1 and IGF-1R in blood vessels (Table 8).

A strong positive correlation was found only between FGFR1 and β-defensin 2 in connective tissue (rs: 0.624; *p*-value: 0.03).

A strong positive correlation was present between Wnt-1 and β-defensin 2 (rs: 0.69; *p*-value: 0.013), Wnt-1 and IGF-1 (rs: 0.649; *p*-value: 0.022), a moderate positive correlation was found between Wnt-1 and TGF-ß (rs: 0.584; *p*-value: 0.046) in mesothelium.

## 4. Discussion

Immunoreactive expression was compared between the patient and the control groups in different diaphragm sites of diaphragm hernia to determine the significance of different growth factors and their receptors, muscle and nerve quality factors, local defense factors, programmed cell death, and separate gene expression in the development of CDH. Histological structures and correlations between immunoreactives were also made.

Although the data showed slight alterations between different sites of diaphragm hernia, no significant changes were determined between these sites, showing either that the immunoreactives are distributed equally in different sites of the diaphragm or these changes are not possible to detect with the number of patients we had in our research.

CDH patient muscle fibers showed variable expression of bFGF and IGF-1 growth factors, IGF-1 had the most notable fluctuations. FGFR1 also presented a variable expression in muscle fibers. Statistically significant changes in bFGF and FGFR expression were noted between the patient and the control group muscle fibers. These growth factors presented strong correlations between IGF-1 and bFGF, FGFR1 and IGF-1, bFGF and IGF-1R, showing that either these growth factors interact with each other or there is a pathway that makes alternations in their expression. For instance, one of such pathways is a retinoid acid pathway and alterations in this pathway are determined as a significant factor for the development of CDH [36]. Furthermore, bFGF and IGF-1 expression can be affected by a retinoid acid pathway [37,38], thus we speculate that immunoreactive alternations in muscle fibers detected in our patients can be caused by alternations in the retinoid acid pathway.

Remarkably, bFGF had no expression in the control group diaphragm tissue whereas the CDH patient muscle fibers showed an unstable expression of bFGF. We assume this to be a complete novelty for this anomaly. A strong correlation between bFGF and FGFR was detected. It was found that with a higher expression of bFGF in the patient tissue, FGFR expression was lower compared to the control group, where bFGF was not expressed at all.

Muscle quality markers showed compelling data. Myosin and α-actin expression in the diaphragm muscle fibers had a stable expression; however, dystrophin presented a variable expression in both the patient and the control group diaphragm muscle fibers. We assume these relative changes in dystrophin expression could be due to the diaphragm development processes involving apoptosis in the muscle mass [39].

Immunoreactive expression in blood vessels had numerous inconsistencies, showing significant changes in TGF-β, FGFR1, IGF-1R, NGF, β-defensin 2 and apoptosis between the patient and the control groups. We suppose these factors could play an important role in the pathogenesis of CDH through the development of blood vessels.

It should be noted that TGF-β signaling pathway is considered important for the proper diaphragm formation [40]. TGF-β expression was slightly higher and had variations in CDH patients comparing the patient and the control group tissue; meanwhile the control group presented a stable expression, suggesting CDH patients could have TGF-β pathway alterations in blood vessels. Significance of the alternations in TGF-β pathway has also been proved by other scientists that studied the development of CDH [36].

Strong intercorrelations in blood vessels were found between FGFR1, IGF-1, IGF-1R and Wnt-1 as well as significant changes in FGFR1, IGF-1R expression between the patient and the control group. As the activation of FGFR1 and IGF-1R receptors can lead to activation of PI3K/Akt pathway [41,42,43], these strong intercorrelations with wnt-1 suggest the presence of synergism between the PI3K/Akt pathway and the wnt/β-catenin pathway. Such synergy was found in Skurk et al. research suggesting that VEGF/PI3-kinase/Akt signaling is a downstream of β-catenin, and that it contributes to the pro-angiogenic actions of β-catenin on endothelial cells [43]. Therefore, fluctuations in Wnt-1 gene expression could cause the significant changes in FGFR1 and IGF-1R expression between the groups.

IGF-1R had a relatively high and stable expression in the diaphragm tissue, with alternations between the patient and the control groups only in blood vessels. This suggests that IGF-1R is not affected in other tissues in the presence of CDH. IGF-1R could be involved in CDH pathogenesis through angiogenetic processes as well as previously mentioned IGF-1R, FGFR1, Wnt pathway interactions.

The evaluation of β-defensin 2 expression revealed that it was higher in the control group than in the CDH patients. According to Baroni et al., research β-defensin 2 has properties to promote endothelial cell proliferation [29]. Although the exact mechanism through which β-defensin 2 exerts its pro-angiogenetic properties is still unclear, it is clearly seen that the various cytokines involved in pro-angiogenetic processes are less expressed in CDH affected blood vessels than in the control group tissue. These findings further accent that there is a dysregulation in angiogenetic processes of the formation of CDH. Angiogenetic processes might have a crucial role in the pathogenesis of CDH.

However, the data shows various cytokines responsible for angiogenetic processes being affected, a more detailed research on the interactions between the above-mentioned pathways and growth factors should be carried out to confirm this theory.

The immunoreactive expression was stable in connective tissue with an alteration between the patient and the control groups in HGF expression with a higher expression in the patient tissue. Overall HGF expression was stable and had a relatively high expression comparing it to other immunoreactives. The studies reveal that muscle cells migrate from somites into the PPFs [21] in the presence of HGF. This shows that HGF should have a significant impact on CDH development; however, no statistically significant changes were found in other diaphragm tissues. We presume alternations in other tissues may be seen in embryonal development. As for an increased HGF expression in the connective tissue, it could be a compensatory mechanism after the development of CDH to increase the number of myocytes in areas where the hernia has not developed and to increase the overall strength of a diaphragm.

Mesothelium had very few alternations in the immunoreactive expression, showing that mesothelium is either slightly or not affected by CDH at all.

β-defensin 4 showed an absence of positive muscle fibers in the proximal and distal parts of the diaphragm and its expression fluctuated from absent to few positive structures in the central part. β-defensin 4 is an anti-microbial agent and its expression increases in response to inflammatory signals. It has been reported that the gene of the human β-defensin 4, called hBD-4, is limited to only a few tissues and the diaphragm is not one of them [30]. The β-defensin levels coincide with the controls and the herniated diaphragms. We can see no bacterial infection therefore we can conclude that in normal condition β-defensin 4 is probably not expressed in diaphragm tissue. β-defensin 2 was expressed in both the patient group and their controls without significant differences. It provided fluctuating results in the CDH patient tissues. As β-defensin genes are close to chromosome 8p23.1, which is a hotspot with genes responsible for CDH formation [44,45], some deletions might include the defensin gene clusters, where variants in the non-deleted allele or total deletion of defensin coding gene might further influence defensin expression in tissues.

There are two existing hypotheses of how CDH occurs. Some investigators voice an opinion of an increased apoptosis [46,47], others—of a decreased cell proliferation [32,48] to be at fault of CDH development. Our findings contradict the increased cell death theory because the number of apoptotic cells in the patient tissue was the same or slightly decreased compared to the number of apoptotic cells in the control group. One of the herniated samples showed a few solitary apoptotic cells in the connective tissue, while all surrounding structures appeared negative showing that there was a high variability of cells affected by apoptosis in the patient tissue. Therefore, we can conclude that CDH can develop without elevated programmed cell death numbers. We can hypothesize that there are different pathways how CDH can be formed, through a high variability of apoptotic cell numbers in CDH patient tissue for instance.

The number of NGF and NGFR positive nerve fibers varied from one nerve bundle to another within each sample. It is notable that NGF and its receptor did not correlate with each other. Still, NGF remained higher in all samples. High NGF numbers have been reported to induce increased levels of matrix metalloproteases (MMPs). MMPs enhance skeletal muscle fiber regeneration by promoting satellite muscle cell migration and differentiation [49]. In this context, MMP-1,-2,-7,-9,-13 have been emphasized [49]. Consequently, NGF expression needs to be elevated in case of the diaphragm injury, but we see that it is not so. In addition, a theory has been proposed that NGF, in fact, inhibits the function of MMPs [50]. Therefore, the decreased number of NGF positive structures could imply to either over-expression or under-expression of MMPs.

NGF is expressed in higher numbers than NGFR, which may suggest that a part of NGF attaches to the TrkA NGFR whose expression was not measured in this study. Other studies say that MMP-2 is activated exactly through TrkA NGFR stimulation, which promotes angiogenesis [51]. In addition, VEGF levels have been found to respond to elevated NGF expression with an increase in numbers, further giving rise to neovascularization [23,52]. We noticed a decrease of NGF in blood vessel walls, which indicates that blood vessel formation in CDH might be impaired.

NGFR guides phrenic nerve growth from neural tube to PPFs. At the same time, p75NGFR when binding to NGF is capable of inducing apoptosis in fibroblasts and myofibroblasts [53]. The marker’s expression is decreased in the nerve bundles and the blood vessel walls of the herniated diaphragm samples, which may have led to an increased fibroblast lifespan that decreased healing abilities of the diaphragmatic defects. This coincides with the reduced number of apoptotic cells visualized with TUNEL.

Wnt-1 demonstrated high expression variability in blood vessels and mesothelium. Its levels of signaling did not correlate with the cell death marker, which might be because Wnt-1 protects cells from apoptosis only when expressed in exceedingly high numbers as in cancer cells [54]. Wnt-1 stabilizes β-catenin, whose loss of function in mouse models leads to diaphragm development defect [34]. We, however, observed that Wnt-1 was up-regulated in most CDH patient endotheliocytes, mesotheliocytes, and muscle fibers, which might be a compensatory readjustment. One hypothesis is that elevated Wnt-1 numbers might be at fault of underdeveloped diaphragm tissue architecture. This explains why Wnt-1 levels in muscle fibers correlate with myosin, which is expressed only in differentiated muscle cells. However, this rise in Wnt-1 numbers alone most likely could not be responsible for CDH development. In Wilms Tumor 1 (Wt-1) knock-out mice models the diaphragms develop without CDH when β-catenin and, probably, Wnt-1 levels are kept elevated [34]. The loss of Twist-related protein 1 (Twist1) positive cells is assumed to be a common factor why CDH develops in reduced Wt-1 and β-catenin pathway models [34]. Both Wt-1 and Wnt-1 influence β-catenin signaling and Twist1 expression [34,55].

## 5. Conclusions

Different tissue factor appearance and distribution seem to correlate with the specific morphopathogenetic mechanism but do not depend on the specific site of affected diaphragm.

With a notable increase of bFGF, FGFR1 expression decreased in patient tissue (supported by a significant intercorrelation between these factors). This reveals a new pathogenetic phenotype for CDH, suggesting the prominent stimulation of growth in mesenchymal origin tissue.

The prominent and stable expression of IGF-1R and HGF and moderate expression of TGF-β show these factors as essential growth stimulators in CDH affected tissue. Intercorrelations between these factors and local defense factors (β-defensin 2, but not 4) and apoptosis indicate the leading role of tissue growth, anti-microbial defense, and programmed cell death in the morphopathogenesis of CDH.

Variable Wnt-1 expression in patient blood vessels, while being stable in controls and having strong intercorrelations with IGF-1, FGFR1, and IGF-1R suggests dysregulation in angiogenetic processes in herniated diaphragms.

The minor changes of muscle quality markers myosin and α-actin expression between the patient and the control groups are less likely to be involved in CDH pathogenesis, while the variable dystrophin expression in both the CDH patient and the controls seems to manifest itself more due to the diaphragm development processes involving apoptosis in muscle mass.

Only mesothelium presents insignificant alternations in different tissue factor expressions throughout the herniated diaphragm tissue, thus suggesting that CDH has almost no effect on mesotheliocytes.

Decreased NGF, NGFR and NF expression in CDH diaphragm indicates the possible decrease of neuronal structure quality in the pathogenesis of this anomaly.

## Figures and Tables

**Table 1:** Semi-quantitative evaluation of the relative number of TGF-β, bFGF, and FGFR1 in the CDH patient and the control groups.

		**TGF-β**				**bFGF**	**FGFR1**			
		Mf	Ct	B	M	Mf	Mf	Ct	B	M
P	Patients	+/++	+	+/++	++/+++	0–0/+	++/+++	+/++–++/+++	++/+++	+++
	Controls	++	+	+/++	++	0	++++	0/+–++	++	+++
C	Patients	++	+/++	++	+	0/+	+++	0/+–++	+++	+++
	Controls	++	0–++	+/++	++	0–0/+	+++	0/+–++	++	+++
D	Patients	++	0/+	++	++	0/+–+++	++/+++	0/+–+++	++	0/+
	Controls	++	0–+	+/++	++	0	+++/++++	+/++	+/++	++/+++

Abbreviations: Mf—muscle fibers; Ct—connective tissue; B—blood vessels; M—mesothelium; P—proximal part of diaphragm; C—central part of diaphragm; D—distal part of diaphragm; TGF-β—Transforming growth factor-beta; bFGF—basic fibroblast growth factor; FGFR1—Fibroblast growth factor receptor 1; 0—no positive structures, 0/+—occasional positive structures, +—few positive structures, +/++—few to moderate number of positive structures, ++—moderate number of positive structures, ++/+++—moderate to numerous positive structures, +++—numerous positive structures, +++/++++—numerous to abundant structures, ++++—abundance of positive structures in the visual field.

**Table 2:** Semi-quantitative evaluation of the relative number of IGF-1, IGF-1R, and HGF in the CDH patient and the control groups.

		**IGF-1**				**IGF-1R**				**HGF**			
		Mf	Ct	B	M	Mf	Ct	B	M	Mf	Ct	B	M
P	Patients	+	++/+++	++	+++	++/+++	+++	++/+++	+++	++/+++	+++	++/+++	+++
	Controls	+++	+++	++++	+++	+++	+++	+++	+++	++/+++	++/+++	++/+++	+++
C	Patients	++/+++	++	++/+++	+++	++/+++	+++	++/+++	+++	+++	+++	++/+++	+++
	Controls	+/++	++	+++	+++	+–+++	+++	+++	+++	++/+++	++/+++	++/+++	+++
D	Patients	++/+++	++/+++	++/+++	+++	++/+++	+++	++/+++	+++	++/+++	+++	+++	+++
	Controls	+	++	++	+++	+++	+++	+++	+++	++/+++	++/+++	++/+++	+++

Abbreviations: Mf—muscle fibers; Ct—connective tissue; B—blood vessels; M—mesothelium; P—proximal part of diaphragm; C—central part of diaphragm; D—distal part of diaphragm; IGF-1—Insulin-like growth factor 1; IGF-1R—Insulin-like growth factor 1 receptor; HGF—Hepatocyte growth factor; 0—no positive structures, 0/+—occasional positive structures, +—few positive structures, +/++—few to moderate number of positive structures, ++—moderate number of positive structures, ++/+++—moderate to numerous positive structures, +++—numerous positive structures, +++/++++—numerous to abundant structures, ++++—abundance of positive structures in the visual field.

**Table 3:** Semi-quantitative evaluation of the relative number of muscle quality factors in the CDH patient and the control groups.

		**Myosin**	**Dystrophin**		**α-Actin**	
		Mf	Mf	B	Mf	B
P	Patients	++/+++	+–+++	+	+–++++	++++
	Controls	++/+++	+–+++	0/+	+++	++++
C	Patients	++/+++	++	0/+	+++	++++
	Controls	++/+++	+–+++	0/+	+++/++++	++++
D	Patients	+–+++	0/+–+++	0/+	+++	++++
	Controls	++/+++	+–+++	0/+	++++	++++

Abbreviations: Mf—muscle fibers; B—blood vessels; P—proximal part of diaphragm; C—central part of diaphragm; D—distal part of diaphragm; 0—no positive structures, 0/+—occasional positive structures, +—few positive structures, +/++—few to moderate number of positive structures, ++—moderate number of positive structures, ++/+++—moderate to numerous positive structures, +++—numerous positive structures, +++/++++—numerous to abundant structures, ++++—abundance of positive structures in the visual field.

**Table 4:** Semi-quantitative evaluation of the relative number of local defense factors and programmed cell death in the CDH patient and the control groups.

		**β-Defensin 2**				**β-Defensin 4**	**Apoptosis**			
		Mf	Ct	B	M	Mf	Mf	Ct	B	M
P	Patients	++	+	0	+++	0	++	+–+++	+–+++	+–++++
	Controls	++	0/+	+	+++	0	++	++/+++	++/+++	++/+++
C	Patients	++	0/+	+	0	0/+	++	+–+++	++	+–++++
	Controls	++	0/+	+/++	0–+++	0/+	++	+++	+++/++++	+++/++++
D	Patients	++	+	0/+	0–+++	0	++	++	+–+++	++/+++
	Controls	++	0/+	+	++/+++	0	++	++	++/+++	++/+++

Abbreviations: Mf—muscle fibers; Ct—connective tissue; B—blood vessels; M—mesothelium; P—proximal part of diaphragm; C—central part of diaphragm; D—distal part of diaphragm; 0—no positive structures, 0/+—occasional positive structures, +—few positive structures, +/++—few to moderate number of positive structures, ++—moderate number of positive structures, ++/+++—moderate to numerous positive structures, +++—numerous positive structures, +++/++++—numerous to abundant structures, ++++—abundance of positive structures in the visual field.

**Table 5:** Semi-quantitative evaluation of the relative number of nerve quality factors in the CDH patient and the control groups.

		**NGF**			**NGFR**			**NF**
		Ct	B	Nf	Ct	B	Nf	Nf
P	Patients	+	+	+	0/+–+	0	0/+	0
	Controls	+	++	++	0/+	+	+++	0–+++
C	Patients	+	+/++	0/+	+	++/+++	0	0–+++
	Controls	+	+–+++	+–+++	0/+–+	0/+–+++	0/+–+++	0–+++
D	Patients	+	0/+	0	0/+–+	++	0	0
	Controls	+/++	++	0/+	0/+–+	++/+++	0/+–+++	0–+++

Abbreviations: NGF—nerve growth factor; NGFR—nerve growth factor receptor; NF—neurofilaments; Ct—connective tissue; B—blood vessels; Nf—nerve fibers; P—proximal part of diaphragm; C—central part of diaphragm; D—distal part of diaphragm; 0—no positive structures, 0/+—occasional positive structures, +—few positive structures, +/++—few to moderate number of positive structures, ++—moderate number of positive structures, ++/+++—moderate to numerous positive structures, +++—numerous positive structures, +++/++++—numerous to abundant structures, ++++—abundance of positive structures in the visual field.

**Table 6:** A semi-quantitative evaluation of the relative number of Wnt-1 gene expression in the CDH patient and the control groups.

		**Wnt-1**			
		Mf	Ct	B	M
P	Patients	+++	0	0/+–+++	0–++++
	Controls	+++	0	+	0
C	Patients	+++/++++	0	0/+–++++	0
	Controls	+++	0	0/+	0
D	Patients	+++/++++	0/+	+/++	0–++++
	Controls	+++	0	+	0

Abbreviations: Mf—muscle fibers; Ct—connective tissue; B—blood vessels; M—mesothelium; P—proximal part of diaphragm; C—central part of diaphragm; D—distal part of diaphragm; 0—no positive structures, 0/+—occasional positive structures, +—few positive structures, +/++—few to moderate number of positive structures, ++—moderate number of positive structures, ++/+++—moderate to numerous positive structures, +++—numerous positive structures, +++/++++—numerous to abundant structures, ++++—abundance of positive structures in the visual field.

**Table 7:** Spearman’s rank correlation coefficient between different tissue factors in the patient group muscle fibers.

Strength of Correlation	Correlations between Immunoreactive Structures in the Patient Group Muscle Fibers	r_s_	*p*-Value
“very strong” 0.8–1.0	TGF-β and FGFR1	0.911	<0.001
“strong” 0.6–0.79	Myosin and apoptosis	0.784	0.003
	HGF and IGF-1R	0.744	0.006
	FGFR1 and IGF-1	0.668	0.018
	bFGF and FGFR1	0.663	0.019
	TGF-β and IGF-1	0.644	0.024
	bFGF and IGF-1R	0.627	0.029
	IGF-1 and bFGF	0.615	0.033
	IGF-1 and β-defensin 4	0.603	0.038
“moderate” 0.4–0.59	TGF-β and bFGF	0.585	0.046

Abbreviations: TGF-β—Transforming growth factor-beta; bFGF—basic fibroblast growth factor; FGFR1—Fibroblast growth factor receptor 1; IGF-1—Insulin-like growth factor 1; IGF-1R—Insulin-like growth factor 1 receptor; HGF—Hepatocyte growth factor, r_s_—Correlation strength.

**Table 8:** Spearman’s rank correlation coefficient correlations between different tissue factors in the patient group blood vessels.

Strength of Correlation	Correlations between Immunoreactive Structures in Patient Group Blood Vessels	r_s_	*p*-Value
“very strong” 0.8–1.0	FGFR1 and IGF-1	0.812	0.001
“strong” 0.6–0.79	HGF and TGF-β	0.786	0.002
	Wnt-1 and IGF-1	0.741	0.006
	Wnt-1 and FGFR1	0.732	0.007
	TGF-β and β-defensin 2	0.708	0.010
	Wnt-1 and IGF-1R	0.704	0.011
	TGF-β and FGFR1	0.695	0.012
	TGF-β and IGF-1	0.650	0.022
	IGF-1 and IGF-1R	0.648	0.023

Abbreviations: TGF-β—Transforming growth factor-beta; bFGF—basic fibroblast growth factor; FGFR1—Fibroblast growth factor receptor 1; IGF-1—Insulin-like growth factor 1; IGF-1R—Insulin-like growth factor 1 receptor; HGF—Hepatocyte growth factor; Wnt-1—wingless gene-1, r_s_—Correlation strength.

## Data Availability

The data presented in this study are available on request from the corresponding author. The data are not publicly available due to ethical considerations and children material used in the study.

## References

[1] McGivern M.R., Best K.E., Rankin J., Wellesley D., Greenlees R., Addor M.-C., Arriola L., de Walle H., Barisic I., Beres J. (2015). Epidemiology of congenital diaphragmatic hernia in Europe: A register-based study. Arch. Dis. Child. Fetal Neonatal Ed..

[2] Merrell A.J., Kardon G. (2013). Development of the diaphragm—a skeletal muscle essential for mammalian respiration. Febs. J..

[3] Alaggio R., Midrio P., Sgrò A., Piovan G., Guzzardo V., Donato R., Sorci G., Lago P., Gamba P.G. (2015). Congenital Diaphragmatic Hernia: Focus on Abnormal Muscle Formation. J. Pediatric Surg..

[4] Wynn J., Yu L., Chung W.K. (2014). Genetic Causes of Congenital Diaphragmatic Hernia. Semin. Fetal Neonatal Med..

[5] Iwashita N., Sakaue M., Shirai M., Yamamoto M. (2018). Early Development of Pleuroperitoneal Fold of the Diaphragm in the Rat Fetus. J. Vet. Med. Sci..

[6] Ornitz D.M., Itoh N. (2015). The Fibroblast Growth Factor signaling pathway. Wiley Interdiscip Rev. Dev. Biol..

[7] Akl M.R., Nagpal P., Ayoub N.M., Tai B., Prabhu S.A., Capac C.M., Gliksman M., Goy A., Suh K.S. (2016). Molecular and clinical significance of fibroblast growth factor 2 (FGF2 /bFGF) in malignancies of solid and hematological cancers for personalized therapies. Oncotarget.

[8] Chae Y.K., Ranganath K., Hammerman P.S., Vaklavas C., Mohindra N., Kalyan A., Matsangou M., Costa R., Carneiro B., Villaflor V.M. (2017). Inhibition of the fibroblast growth factor receptor (FGFR) pathway: The current landscape and barriers to clinical application. Oncotarget.

[9] Lieu C., Heymach J., Overman M., Tran H., Kopetz S. (2011). Beyond VEGF: Inhibition of the Fibroblast Growth Factor Pathway and Antiangiogenesis. Clin. Cancer Res..

[10] Kubiczkova L., Sedlarikova L., Hajek R., Sevcikova S. (2012). TGF-β—An Excellent Servant but a Bad Master. J. Transl. Med..

[11] Xu P., Liu J., Derynck R. (2012). Post-translational regulation of TGF-β receptor and Smad signaling. Febs. Lett..

[12] Hill C.S. (2016). Transcriptional Control by the SMADs. Cold Spring Harb. Perspect Biol..

[13] Qin Z., Worthen C.A., Quan T. (2017). Cell-size-dependent upregulation of HGF expression in dermal fibroblasts: Impact on human skin connective tissue aging. J. Derm. Sci..

[14] Guerrero P.A., McCarty J.H. (2017). TGF-β Activation and Signaling in Angiogenesis. Physiol. Pathol. Angiogenesis Signal. Mech. Target. Ther..

[15] Yakar S., Adamo M.L. (2012). Insulin-Like Growth Factor-1 Physiology: Lessons from Mouse Models. Endocrinol. Metab Clin. North. Am..

[16] Fong K.M., Larsen J.E., Wright C., Sriram K., Davidson M., Daniels M., Sekido Y., Bowman R.V., Yang I.A., Minna J.D. (2016). Molecular basis of lung carcinogenesis. Mol. Basis Hum. Cancer.

[17] Shuang T., Fu M., Yang G., Wu L., Wang R. (2018). The interaction of IGF-1/IGF-1R and hydrogen sulfide on the proliferation of mouse primary vascular smooth muscle cells. Biochem. Pharm..

[18] Delafontaine P., Song Y.-H., Li Y. (2004). Expression, regulation, and function of IGF-1, IGF-1R, and IGF-1 binding proteins in blood vessels. Arter. Thromb. Vasc. Biol..

[19] Lin S., Zhang Q., Shao X., Zhang T., Xue C., Shi S., Zhao D., Lin Y. (2017). IGF-1 Promotes Angiogenesis in Endothelial Cells/Adipose-Derived Stem Cells Co-Culture System with Activation of PI3K/Akt Signal Pathway. Cell Prolif..

[20] Mungunsukh O., McCart E.A., Day R.M. (2014). Hepatocyte Growth Factor Isoforms in Tissue Repair, Cancer, and Fibrotic Remodeling. Biomedicines.

[21] Mitchell R.N., Kumar V., Abbas A.K., Aster J.C. (2014). The Cell as a Unit of Health and Disease. Robbins and Cotran Pathologic Basis of Disease.

[22] Ameis D., Khoshgoo N., Keijzer R. (2017). Abnormal lung development in congenital diaphragmatic hernia. Semin. Pediatr. Surg..

[23] Skaper S.D. (2017). Nerve growth factor: A neuroimmune crosstalk mediator for all seasons. Immunology.

[24] Wheeler E., Bothwell M. (1992). Spatiotemporal patterns of expression of NGF and the low-affinity NGF receptor in rat embryos suggest functional roles in tissue morphogenesis and myogenesis. J. Neurosci..

[25] Wong V., Arriaga R., Ip N.Y., Lindsay R.M. (1993). The neurotrophins BDNF, NT-3 and NT-4/5, but not NGF, up-regulate the cholinergic phenotype of developing motor neurons. Eur. J. Neurosci..

[26] Domeniconi M., Hempstead B.L., Chao M.V. (2007). Pro-NGF secreted by astrocytes promotes motor neuron cell death. Mol. Cell Neurosci..

[27] Wang H., Wu M., Zhan C., Ma E., Yang M., Yang X., Li Y. (2012). Neurofilament proteins in axonal regeneration and neurodegenerative diseases. Neural Regen Res..

[28] Dorin J.R., McHugh B.J., Cox S.L., Davidson D.J., Tang Y.-W., Sussman M., Liu D., Poxton I., Schwartzman J. (2015). Chapter 30—Mammalian Antimicrobial Peptides; Defensins and Cathelicidins. Molecular Medical Microbiology.

[29] Baroni A., Donnarumma G., Paoletti I., Longanesi-Cattani I., Bifulco K., Tufano M.A., Carriero M.V. (2009). Antimicrobial Human Beta-Defensin-2 Stimulates Migration, Proliferation and Tube Formation of Human Umbilical Vein Endothelial Cells. Peptides.

[30] García J.-R.C., Krause A., Schulz S., Rodríguez-Jiménez F.-J., Klüver E., Adermann K., Forssmann U., Frimpong-Boateng A., Bals R., Forssmann W.-G. (2001). Human β-defensin 4: A novel inducible peptide with a specific salt-sensitive spectrum of antimicrobial activity. Faseb J..

[31] Ni D., Mb B. (2007). Osmotic stress and DNA damage. Methods Enzym..

[32] Clugston R.D., Zhang W., Greer J.J. (2010). Early development of the primordial mammalian diaphragm and cellular mechanisms of nitrofen-induced congenital diaphragmatic hernia. Birth Defects Res. A Clin. Mol. Teratol..

[33] Mudri M. (2018). The Effects of Tracheal Occlusion on Wnt Signaling in a Rabbit Model of Congenital Diaphragmatic Hernia. Electron. Thesis Diss. Repos..

[34] Paris N.D., Coles G.L., Ackerman K.G. (2015). Wt1 and β-Catenin Cooperatively Regulate Diaphragm Development in the Mouse. Dev. Biol..

[35] Pilmane M., Shine J., Iismaa T.P. (1998). Distribution of Galanin Immunoreactivity in the Bronchi of Humans with Tuberculosis. Ann. N. Y. Acad. Sci..

[36] Russell M.K., Longoni M., Wells J., Maalouf F.I., Tracy A.A., Loscertales M., Ackerman K.G., Pober B.R., Lage K., Bult C.J. (2012). Congenital diaphragmatic hernia candidate genes derived from embryonic transcriptomes. Proc. Natl. Acad. Sci. USA.

[37] Gaetano C., Catalano A., Illi B., Felici A., Minucci S., Palumbo R., Facchiano F., Mangoni A., Mancarella S., Mühlhauser J. (2001). Retinoids induce fibroblast growth factor-2 production in endothelial cells via retinoic acid receptor alpha activation and stimulate angiogenesis in vitro and in vivo. Circ Res..

[38] Fu Z., Noguchi T., Kato H. (2001). Vitamin A deficiency reduces insulin-like growth factor (IGF)-I gene expression and increases IGF-I receptor and insulin receptor gene expression in tissues of Japanese quail (Coturnix coturnix japonica). J. Nutr..

[39] Dupont-Versteegden E.E. (2006). Apoptosis in skeletal muscle and its relevance to atrophy. World J. Gastroenterol.

[40] Bielinska M., Jay P.Y., Erlich J.M., Mannisto S., Urban Z., Heikinheimo M., Wilson D.B. (2007). Molecular genetics of congenital diaphragmatic defects. Ann. Med..

[41] Nakashio A., Fujita N., Tsuruo T. (2002). Topotecan inhibits VEGF- and bFGF-induced vascular endothelial cell migration via downregulation of the PI3K-Akt signaling pathway. Int. J. Cancer.

[42] Chen G.J., Weylie B., Hu C., Zhu J., Forough R. (2007). FGFR1/PI3K/AKT signaling pathway is a novel target for antiangiogenic effects of the cancer drug fumagillin (TNP-470). J. Cell Biochem..

[43] Carsten S., Henrike M., Edward R., Ann B., Thomas F. (2005). Walsh Kenneth Glycogen-Synthase Kinase3β/β-Catenin Axis Promotes Angiogenesis Through Activation of Vascular Endothelial Growth Factor Signaling in Endothelial Cells. Circ. Res..

[44] Longoni M., Lage K., Russell M.K., Loscertales M., Abdul-Rahman O.A., Baynam G., Bleyl S.B., Brady P.D., Breckpot J., Chen C.P. (2012). Congenital diaphragmatic hernia interval on chromosome 8p23.1 characterized by genetics and protein interaction networks. Am. J. Med. Genet. A.

[45] Chen C.-P., Peng C.-R., Chang T.-Y., Guo W.-Y., Chen Y.-N., Wu P.-S., Town D.-D., Wang W. (2015). Prenatal diagnosis of chromosome 8p23.1 microdeletion by array comparative genomic hybridization using uncultured amniocytes in a pregnancy associated with fetal partial corpus callosum agenesis and schizencephaly. Taiwan J. Obs. Gynecol..

[46] Alles A.J., Losty P.D., Donahoe P.K., Manganaro T.F., Schnitzer J.J. (1995). Embryonic cell death patterns associated with nitrofen-induced congenital diaphragmatic hernia. J. Pediatr. Surg..

[47] Jay P.Y., Bielinska M., Erlich J.M., Mannisto S., Pu W.T., Heikinheimo M., Wilson D.B. (2007). Impaired mesenchymal cell function in Gata4 mutant mice leads to diaphragmatic hernias and primary lung defects. Dev. Biol..

[48] Fisher J.C., Bodenstein L. (2006). Computer simulation analysis of normal and abnormal development of the mammalian diaphragm. Biol. Med. Model..

[49] Chen X., Li Y. (2009). Role of matrix metalloproteinases in skeletal muscle. Cell Adh Migr.

[50] Wijeyewickrema L.C., Gardiner E.E., Gladigau E.L., Berndt M.C., Andrews R.K. (2010). Nerve Growth Factor Inhibits Metalloproteinase-Disintegrins and Blocks Ectodomain Shedding of Platelet Glycoprotein VI. J. Biol. Chem..

[51] Park M.-J., Kwak H.-J., Lee H.-C., Yoo D.-H., Park I.-C., Kim M.-S., Lee S.-H., Rhee C.H., Hong S.-I. (2007). Nerve growth factor induces endothelial cell invasion and cord formation by promoting matrix metalloproteinase-2 expression through the phosphatidylinositol 3-kinase/Akt signaling pathway and AP-2 transcription factor. J. Biol. Chem..

[52] Diao Y.-P., Cui F.-K., Yan S., Chen Z.-G., Lian L.-S., Guo L.-L., Li Y.-J. (2016). Nerve Growth Factor Promotes Angiogenesis and Skeletal Muscle Fiber Remodeling in a Murine Model of Hindlimb Ischemia. Chin. Med. J. (Engl.).

[53] Micera A., Puxeddu I., Balzamino B.O., Bonini S., Levi-Schaffer F. (2012). Chronic Nerve Growth Factor Exposure Increases Apoptosis in a Model of In Vitro Induced Conjunctival Myofibroblasts. PLoS ONE.

[54] Deitrick J., Pruitt W.M. (2016). Wnt/β Catenin-Mediated Signaling Commonly Altered in Colorectal Cancer. Prog. Mol. Biol. Transl. Sci..

[55] Reinhold M.I., Kapadia R.M., Liao Z., Naski M.C. (2006). The Wnt-inducible transcription factor Twist1 inhibits chondrogenesis. J. Biol. Chem..

